# The impact of inpatient bed capacity on length of stay

**DOI:** 10.1007/s10198-021-01373-2

**Published:** 2021-09-04

**Authors:** Brendan Walsh, Samantha Smith, Maev-Ann Wren, James Eighan, Seán Lyons

**Affiliations:** 1 Economic and Social Research Institute, Whitaker Square, Sir John Rogerson’s Quay, Dublin 2, Dublin, Ireland; 2 Department of Economics, Trinity College Dublin, Dublin, Ireland; 3 Public Health and Primary Care, Trinity College Dublin, Dublin, Ireland

**Keywords:** Hospital behaviour, Length of stay, Bed capacity, Emergency care, I10, I18

## Abstract

**Objective:**

Large reductions in inpatient length of stay and inpatient bed supply have occurred across health systems in recent years. However, the direction of causation between length of stay and bed supply is often overlooked. This study examines the impact of changes to inpatient bed supply, as a result of recession-induced healthcare expenditure changes, on emergency inpatient length of stay in Ireland between 2010 and 2015.

**Study design:**

We analyse all public hospital emergency inpatient discharges in Ireland from 2010 to 2015 using the administrative Hospital In-Patient Enquiry dataset. We use changes to inpatient bed supply across hospitals over time to examine the impact of bed supply on length of stay. Linear, negative binomial, and hospital–month-level fixed effects models are estimated.

**Results:**

U-shaped trends are observed for both average length of stay and inpatient bed supply between 2010 and 2015. A consistently large positive relationship is found between bed supply and length of stay across all regression analyses. Between 2010 and 2012 while length of stay fell by 6.4%, our analyses estimate that approximately 42% (2.7% points) of this reduction was associated with declines in bed supply.

**Conclusion:**

Changes in emergency inpatient length of stay in Ireland between 2010 and 2015 were closely related to changes in bed supply during those years. The use of length of stay as an efficiency measure should be understood in the contextual basis of other health system changes. Lower length of stay may be indicative of the lack of resources or available bed supply as opposed to reduced demand for care or the shifting of care to other settings.

**Supplementary Information:**

The online version contains supplementary material available at 10.1007/s10198-021-01373-2.

## Introduction

There have been significant reductions in hospital length of stay over time across health systems. Between 2000 and 2017, length of stay fell by almost 15% on average (7.4 days in 2000 to 6.3 days in 2014) in EU28 countries [1]. These reductions in inpatient length of stay are due to several factors. Within hospitals, greater use of more efficient surgery [2–4], better discharge planning [5, 6], palliative care planning [7], reductions in delayed discharges [8, 9], and more efficient payment mechanisms [10–13] have been shown to reduce length of stay. For specific patient populations, focused care units, such as for early supported discharge units and stroke units [14, 15], and greater provision of non-acute follow-up and rehabilitation care [8, 16–18] has allowed patients to be discharged more quickly. This long, but not exhaustive, list illustrates the many mechanisms that policymakers can use to manage inpatient length of stay.

Lower length of stay is often used as an indicator of efficient care. Intuitively, a shorter length of stay involves less use of health system resources to treat a given case. However, the usefulness of reduced length of stay as an efficiency metric depends upon the assumption that quality of care is unchanged. This is not necessarily so, and whether it is true in a particular context may depend upon why length of stay has fallen. For example, lower length of stay may also be indicative of a lack of resources or reduced bed supply, with subsequent negative consequences for patients.

The extent to which reduced length of stay reduces acute capacity requirements, or vice versa, is a complex and nuanced question. Developed countries have seen a sharp reduction in inpatient bed supply in recent years [19] (Appendix Figure A1). While lower demand for inpatient care, and better use of non-acute care, may explain some of the reduction in beds per capita, other events such as economic shocks (e.g. the Great Recession), have been found to contribute to cuts to healthcare expenditure including acute bed reductions. As a consequence of the Great Recession, many countries experienced cuts to public healthcare budgets [20] with countries most impacted by the crisis, such as Ireland, enacting severe cuts to public healthcare expenditure [21–24].^1^ Studies that have examined the responses of healthcare systems to changes in resources (e.g. due to a funding shock) hypothesise that health systems and/or hospitals may respond in three specific ways:

1. By increasing occupancy rates, reducing length of stay, and/or decreasing the number of admissions [26];
2. Through greater emphasis on substituting care away from hospitals [27]. However, in circumstances of dramatic funding shocks, the ability for substitution is severely curtailed; and
3. Reductions in acute care services and staff. This is the response we examine in the present study.

We treat changes in inpatient bed supply that occurred as a result of the recession-induced healthcare expenditure cuts in Ireland as exogenous system shocks, allowing us to examine the impact that changes in bed supply can have on inpatient length of stay.

### Hospital care in Ireland

Ireland has a mixed public/private healthcare system. Unlike other European health systems, not all individuals are eligible for free public primary and secondary care [28]. There are two broad categories of eligibility for free public healthcare. Approximately 36% have a Medical Card (public health insurance) that confers free public healthcare. Medical Cards are means-tested, so the poorest in the population are not liable for out-of-pocket (OOP) payments for primary (e.g. GP) or acute public hospital care. Those without a Medical Card pay OOP for public healthcare, though approximately 10% receive free GP care through a GP Visit Card. OOP payments can be high, with a GP visit costing over €50 [28], outpatient and Emergency Department (ED) visits costing €100 (without a GP referral) per attendance, and an inpatient stay costing €80 per night (up to a maximum of €800 per annum). A majority of individuals without a Medical Card purchase private health insurance which mainly covers private hospital care.

There are 50 acute care publicly financed hospitals in Ireland [29], with 29 of these being “Model 3 or 4” hospitals with full-time ED, and 19 for-profit hospitals [30]. Private hospitals operate in parallel to public hospitals, and consultants (senior clinicians) often work across both sectors. Most care provided in private hospitals relates to day-case, outpatient, or elective inpatient care, and important for this study, virtually all emergency care is in public hospitals regardless of the patient’s Medical Card or insurance status [31].

### Economic shocks and public healthcare expenditure in Ireland

Ireland was severely impacted by the Great Recession. Between December 2010 and December 2013, Ireland availed of an economic adjustment programme in which financial aid was provided by the European Commission, European Central Bank, and the International Monetary Fund (the ‘Troika’). Public expenditure was cut across all sectors, and these cuts began prior to the bailout. Between 2007 and 2010, public health expenditure fell by 12 per cent (€15.5bn–€13.3bn) and remained relatively stable until 2016 [32]. Large increases in spending have occurred post-2016.

Pay and pensions account for a large proportion of healthcare expenditure—up to 70% in the acute hospital sector [31]. As a consequence, initially much of the expenditure reduction was achieved through reductions in this category of expenditure [21, 24]. There was an incentivised early retirement scheme [21] and a moratorium on recruitment and promotions [24].^2^ Between 2008 and 2014, over 12,000 staff whole time equivalents (WTEs) were cut from the public health service [23], with a 10 per cent reduction in acute nursing WTEs between 2008 and 2012 alone^3^ (HSE Performance Reports 2008–2012). There were sharp reductions in the supply of inpatient beds. As economic conditions improved from 2013, the moratorium in staffing recruitment was lifted and staffing numbers and supply increased slightly to 2016, with substantial increases occurring post-2016.

**Fig. 1:**
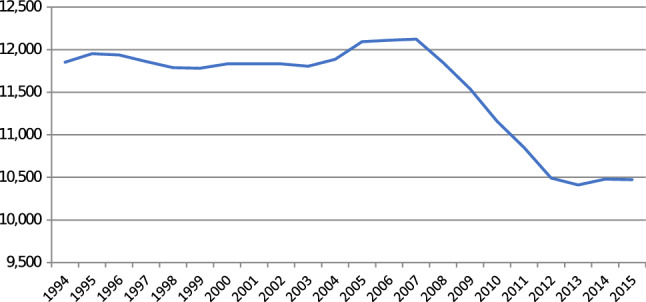
Number of inpatient beds in public hospitals in Ireland: 1994–2015

The cuts to staffing numbers from 2007 onwards resulted in severe reductions in inpatient bed supply during the economic crisis period. As shown in Fig. 1, public hospital inpatient bed supply remained largely stable from the early 1990s until the beginning of the economic crisis, and there was then a marked drop in available inpatient beds of over 13% between 2007 and 2012. A slight increase in inpatient bed availability occurred from 2013 onwards as public healthcare expenditure increased. The cuts to bed supply and staffing should also be seen in the context of a population increasing by 31% between 1996 and 2016 [33]. An additional consequence of the cuts to bed supply, and increased population, is that inpatient bed occupancy rose considerably since 2010, and is the highest in Europe at 95% (Appendix Table A1).

### Study question

This study uses the changes in bed supply between 2010 and 2015 to estimate what impact variations in bed supply may have on emergency inpatient length of stay. We argue that the reductions in bed supply that occurred from 2008 onwards were a result of recession-induced cuts to public healthcare expenditure, with the subsequent improvement in economic conditions from 2013 permitting increases in public health spending that led to increased hiring of staff and re-opening of closed beds. We take advantage of the fact that bed supply changes occurred at different times, and to different degrees, across hospitals. While these analyses have clear implications for policymakers in Ireland, the findings also raise questions about the appropriateness of using inpatient length of stay as an efficiency indicator, particularly when changes in length of stay are understood without taking account of wider changes in the health system.

## Data

### Hospital patient data

Data on length of stay in acute public hospitals were taken from the Hospital In-Patient Enquiry (HIPE) dataset. HIPE is a routinely collected administrative dataset, collected from all public acute hospitals in Ireland. HIPE includes a range of information at the level of discharge including date of admission and discharge, disease classification, health procedure, and a range of patient-level characteristics. For all discharges, information on up to 20 diagnosis codes and, where applicable, 20 procedure codes is recorded.

HIPE is similar to other routinely collected administrative datasets such as Hospital Episode Statistics (HES) from England. However, HIPE lacks a unique patient identifier. This prevents patients’ healthcare utilisation being followed across hospitals and over time. Thus, analyses in this study are conducted at discharge level, rather than at the patient level.

### Inpatient bed data

Data on hospital bed supply were provided by the Health Service Executive (HSE). These data provide the average monthly number of regularly maintained and staffed acute inpatient beds available in acute public hospitals between 2010 and 2015 (there is a lack of a consistent hospital-level series pre-2010). Average supply in each month was calculated by summing all 7-day and 5-day acute beds (beds not open at the weekend) in each hospital in every month and dividing this number by the days in the relevant month. These data are used by the HSE, the Department of Health, and the OECD as reliable estimates of available hospital supply in Irish public hospitals.

### Outcome measures

The dependent variable of interest in this study is emergency inpatient length of stay. Within HIPE, length of stay refers to the time, expressed in days, between admission to and discharge from hospital.

### Independent variables

Inpatient bed supply in public hospitals is the key independent variable included in this study. To compare changes in bed supply in a consistent way across hospitals of different sizes, we constructed a standardised supply variable for each hospital as follows:

1 $${\text{Beds}}\_{\text{St}}_{ht} = \frac{{{\text{Beds}}_{ht} - \overline{{{\text{Beds}}}}_{h} }}{{\sqrt {\frac{1}{N}\mathop \sum \nolimits_{t = 1}^{N} \left( {{\text{Beds}}_{ht} - \overline{{{\text{Beds}}}}_{h} } \right)^{2} } }} ,$$

where the mean number of inpatient beds available in each hospital over the whole period, ${\overline{\mathrm{Beds}} }_{h},$ was subtracted from the number of available inpatient beds in each month, ${\mathrm{Beds}}_{ht}$, and subsequently divided by the standard deviation in bed availability in each hospital over the whole period. This standardised variable has mean equal to zero and standard deviation equal to 1.

Other data included in this study seen to be pertinent to length of stay were: day of admission, year of discharge (which equals year of admission for over 98% of the sample), admission source (home, long-stay facility, transfer from another hospital, other), discharge destination (home, long-stay facility, died, transfer to another hospital, other), age, sex, number of diagnoses, weighted Charlson comorbidity score, marital status, specific Diagnosis-Related Group (DRG),^4^ and Medical Card status.

### Sample

After excluding maternity care, and a small number of discharges that had international addresses or were of no fixed abode (*n* = 12,659), we include 2,237,026 emergency inpatient hospital discharges in HIPE between 2010 and 2015.

Analyses were limited to emergency inpatient admissions for several reasons. First, no corresponding information is available on inpatients in private hospitals. Therefore, it is much more difficult to examine elective inpatient care as many patients can substitute public hospital care (where waiting lists exist) with private hospital care. This is not the case with emergency admissions where the vast majority of emergency activity, particularly complex cases, is undertaken in public hospitals, irrespective of private health insurance coverage status. Second, elective procedures are increasingly provided as day-patient or outpatient procedures, making it difficult to examine changes in length of stay over time. The composition of the elective inpatient category is affected by variations in categories of care on which we do not have data. Third, it is easier to curtail elective care when bed supply declines, but much more difficult to do that for emergency inpatient care. In addition, emergency patients receive priority access to available beds. This means reductions in the probability of admission to a bed are less of a concern, and length of stay in this case will be a better measure of healthcare use.

Since 2005, there has been significant restructuring of the Irish public hospital system. Many hospitals lost Tier 1 ED status, i.e. many EDs were downgraded and were no longer open 24 h per day, 7 days per week. There are 26 adult hospitals with “Model 3 or 4” hospitals with Tier 1 EDs during the 2010–2015 period. To try to avoid biases that might arise due to hospital reconfigurations, analyses were also undertaken on the smaller hospital sub-sample. Tier 1 ED hospitals accounted for 91% of emergency discharges between 2010 and 2015. While most emergency inpatients are admitted from an ED, a small number are admitted from other facilities such as Acute Medical Assessment Units (AMAUs). Patients admitted from AMAUs may have been admitted from another hospital within the larger hospital’s group (and therefore potentially leads to duplicates within the data), or may have already begun their treatment during their AMAU stay and were excluded from the Tier 1 ED analyses, as were readmissions. These criteria result in a sample of 1,484,253 emergency inpatient hospital discharges in the Tier 1 ED analyses.

### Identification strategy

Identifying the causal relationship bed supply may have with length of stay is difficult in the absence of a clear natural experiment. We argue that changes in bed supply between 2010 and 2015 were primarily a result of exogenous shocks to the health system (economic recession and subsequent improvement) and were not related to reduced demand for inpatient care. The economic recession also affected non-acute care provision, and reductions in bed supply at the onset of the recession were not accompanied by major increases in non-acute supply care to allow for planned substitution away from hospital care.

**Fig. 2:**
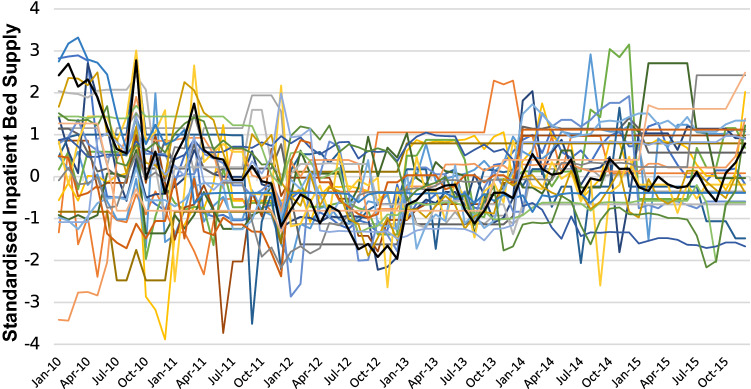
Standardised inpatient bed supply hospital, 2010–2015

The standardised bed supply measure we include allows us to examine changes in relative bed supply at the hospital level and also to compare relative changes across hospitals of different sizes. Figure 2 shows that the fluctuations in bed supply differed across hospitals. While the national average (black line) saw a reduction to December 2012 followed by an increase thereafter, not all hospitals followed the same trajectory. Some hospitals saw reductions (increases) at earlier (later) points in time than others. Therefore, examining changes in standardised inpatient bed supply will not simply capture a time effect, or national trend. The extent of changes in supply differed across hospitals, with some hospitals seeing much greater volatility in supply than others; in other words, variation is observed across hospitals. Therefore, within our analyses, we are comparing hospitals, at the same point in time, with different relative inpatient bed supply. This allows us to better test for the role of bed supply changes on length of stay.

The argument that bed supply reductions were a result of budget constraints rather than improvements in length of stay efficiency has also been borne out from recognition by policymakers of the need for considerable investment in acute bed supply [34]. A health capacity review in 2018 stated that up to 50% more inpatient beds would be required by 2031 to cater for low capacity (Department of Health, 2018a). This lack of capacity also been a clear issue during the COVID-19 pandemic. Waiting lists for elective care are amongst the highest in developed countries (Siciliani et al. 2014), and an Independent Expert Review has also found that significant issues exist with delayed discharges, with a large proportion of delays caused by inadequate non-acute care (Department of Health 2018b, Walsh et al. 2020). These support the view that the changes in inpatient bed supply in recent years were a consequence of exogenous financial shocks, rather than a reduction in demand or better access to non-acute care.

## Methods

We estimate both OLS and negative binomial fixed effects regressions with length of stay (LOS) included at the discharge level.

2 $${\text{LOS}}_{iht} = \alpha Z_{iht} + \gamma H_{ht} + \beta {\text{Beds}}\_{\text{St}}_{ht} + \eta + \tau + \varepsilon_{i} ,$$

where ${\mathrm{LOS}}_{iht}$ is length of stay, for discharge *i*, in hospital *h*, in month *t*. ${Z}_{iht}$ is a vector of discharge-level characteristics. ${H}_{ht}$ are hospital-level characteristics. ${\mathrm{Beds}\_\mathrm{St}}_{ht}$ is the standardised inpatient bed availability in each hospital in month *t*. Hospital fixed effects, $\eta$, and year fixed effects, $\tau$, are included in all analyses.^5^ Within the OLS analysis, length of stay is included in its natural logarithm form, with length of stay included as a count within the negative binomial regression.^6^ As length of stay is heavily skewed to the right (Appendix Figure A2) and influenced by a small number of outliers, the top 1% of the distribution is trimmed. Clustered standard errors at the level of the hospital are estimated.

We also estimate OLS regressions at the hospital–month level:

3 $$\overline{{{\text{LOS}}}}_{ht} = \alpha Z_{ht} + \gamma H_{ht} + \beta {\text{Beds}}\_{\text{St}}_{ht} + {\rm H} + \tau + \upsilon_{ht} ,$$

where ${\overline{\mathrm{LOS}} }_{ht}$ is the mean length of stay for emergency inpatients in hospital *h*, in month *t*. ${Z}_{ht}$ is a measure of patient case-mix. Undertaking the analysis at the hospital–month level reduces the granularity of patient case-mix indicators. Therefore, we follow previous work [36, 37] that includes the age and gender composition of discharges in each hospital–month period as controls for patient case-mix. Additionally, we also include the mean proportion of discharges with Medical Cards, the mean number of diagnoses per discharge, and the mean-weighted Charlson score to further control for patient case-mix, socioeconomic status, and case severity. ${\mathrm{Beds}\_\mathrm{St}}_{ht}$ is a measure of the standardised inpatient bed availability in each hospital in month *t.* Once more hospital and year fixed effects are included. Hospital–months with less than 100 total emergency discharges are excluded. Despite the less granular information on patient case-mix, this model allows for much of the unobserved differences across hospitals to be stripped out and we can assess the net effect of bed supply on length of stay. Length of stay is included in its natural logarithmic form. Clustered standard errors at the level of the hospital are estimated.

## Results

### Descriptive statistics

Table 1 presents descriptive statistics of the discharge sample included in this analysis. Overall, there were over 2.2 million (non-maternity) emergency inpatient discharges in public hospitals between 2010 and 2015. Over 90% of discharges took place in Tier 1 ED hospitals. Average length of stay over this period was 6.4 days. The average age was 49.6 years, 58.6% of discharges had a Medical Card, and discharges had an average of 3.9 diagnoses. Admissions directly from an ED accounted for 73% of discharges, and 85.7% were discharged home.

**Table 1:** Emergency inpatient discharges: 2010–2015

	Number of discharges	
Number of emergency inpatient discharges	2,237,026	
Year of discharge		
2010	339,994	
2011	343,294	
2012	375,414	
2013	383,411	
2014	394,963	
2015	399,950	
	**Mean**	**SD**
Length of stay	6.42	15.56
Tier 1 ED hospital discharges	0.91	–
Age	49.64	27.60
Medical card	0.586	–
Number of diagnoses	3.93	3.19
Weighted Charlson score	0.78	1.63
Mode of emergency admission		
Emergency department	0.732	–
AMAU	0.166	–
Other	0.102	–
Readmission	0.012	0.109
Emergency admissions per hospital per month	1174	513
Discharge destination		
Home	0.852	–
Long stay	0.054	–
Transfer	0.055	–
Died	0.026	–
Other	0.013	–
Admission day		
Sunday	0.096	–
Monday	0.156	–
Tuesday	0.166	–
Wednesday	0.161	–
Thursday	0.157	–
Friday	0.157	–
Saturday	0.107	–
Marital status		
Single/widowed/separated/other	0.626	–
Married	0.374	–

In the 2011–2015 period, only 17.6% of discharges reported a named health insurer

^**†**^Proportion of the sample in each category

**Fig. 3:**
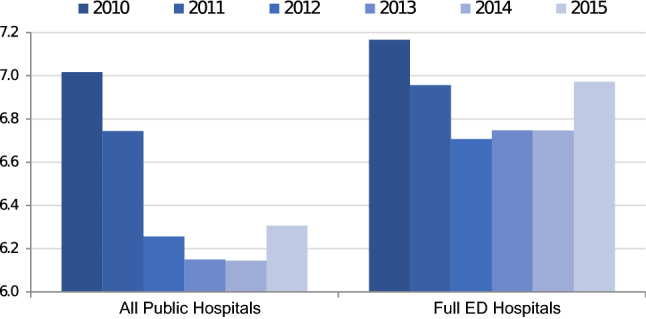
Average length of stay for emergency inpatient discharges: 2010–2015

Figure 3 illustrates the average length of stay for emergency inpatient discharges in all public hospitals and Tier 1 ED hospitals separately. There is some evidence of a U-shape in the average length of stay over time, with average length of stay falling between 2010 and 2012 by 6% in the overall sample and 6.2% in the Tier 1 ED sample, respectively. Between 2012 and 2015 an increase is observed, though length of stay in 2015 is lower than at the beginning of the period. There is evidence that the length of stay increase reported in 2015 was not an anomaly, with average inpatient length of stay in subsequent years remaining flat or continuing to increase slightly.^7^

### Estimation results

Table 2 presents the determinants of length of stay from the discharge-level models. Results show a positive and statistically significant relationship between inpatient bed supply and length of stay in all models. To provide some interpretation for these results, in the Tier 1 ED hospital sample (columns III and VI) a one standard deviation reduction in bed supply implies a reduction in average length of stay of approximately 1.1%. In Tier 1 ED hospitals, the bed supply between 2010 and 2012 fell by 2.6 standard deviations (642 beds), implying a drop in average length of stay of 2.7%. This compares with the 6.4% drop in length of stay actually observed between 2010 and 2012. Results from all public hospitals imply a drop in average length of stay of 6.5% compared to the 10.8% drop actually observed between 2010 and 2012.

**Table 2:** Determinants of length of stay for emergency inpatient discharges (discharge-level model): 2010–2015

	Ordinary least squares (Log Length of Stay)			Negative binomial (Length of Stay)		
	(I)	(II)	(III)	(IV)	(V)	(VI)
Standardised inpatient bed supply	0.018***	0.010***	0.010***	0.018***	0.011***	0.011***
Medical card	0.059**	0.059**	0.055**	0.079***	0.076***	0.072***
Discharge destination						
Home (ref.)						
Long stay	0.766***	0.768***	0.383***	0.885***	0.827***	0.834***
Died	0.116***	0.047***	− 0.003	0.400***	0.362***	− 0.329***
Transfer	0.082**	0.035	0.110***	0.270***	0.233***	0.297***
Other	− 0.189***	− 0.211***	− 0.203***	− 0.083	− 0.078	− 0.086
Tier 1 EDs only	No	No	Yes	No	No	Yes
Readmissions	Yes	No	No	Yes	No	No
Hospital FEs	Yes	Yes	Yes	Yes	Yes	Yes
Clusters	38	38	26	38	38	26
Observations	2,216,733	1,975,782	1,484,253	2,216,733	1,975,782	1,484,253

All models control for age, age squared, sex, weighted Charlson comorbidity index (linear and squared), marital status, day of admission, year of discharge, admission source, DRG, and year fixed effects

Standard errors are clustered at the level of the hospital

^*^p < 0.01, **p < 0.05, ***p < 0.01

Table 2 also indicates that patients with a medical card had 5.5–7.2% longer length of stay, even after controlling for confounders such as age, number of diagnoses, and area of residence. Patients who were discharged to long-stay facilities had much longer length of stay.

Table 3 presents the determinants of length of stay from the hospital-level model. Results show a positive and statistically significant relationship between inpatient bed availability and average length of stay in all models. Results are similar to those shown in Table 2. In column III, the model predicts a 2.7% reduction in length of stay between 2010 and 2012 in the Tier 1 ED hospital sample compared to the 6.4% reduction that actually occurred.

**Table 3:** Determinants of length of stay for emergency inpatient discharges (hospital-level model): 2010–2015

	Ordinary least squares (Log Length of Stay)		
	(I)	(II)	(III)
Standardised inpatient bed supply	0.019***	0.013***	0.013**
Mean medical card	0.042	− 0.168**	− 0.148***
Mean weighted Charlson	0.379***	0.238***	0.234***
Tier 1 ED only	No	No	Yes
Readmissions	Yes	No	No
Hospital FEs	Yes	Yes	Yes
Clusters	38	38	26
Observations	2505	2480	1867

All models control for the age/gender composition of discharges, mean number of diagnoses, total emergency cases by hospital/month, and year fixed effects

Standard errors are clustered at the level of the hospital

Hospital/month periods with less than 100 total emergency discharges are excluded

Discharges with the longest 1% of LOS are excluded: > 60 days

^*^p < 0.01, **p < 0.05, ***p < 0.01

**Fig. 4:**
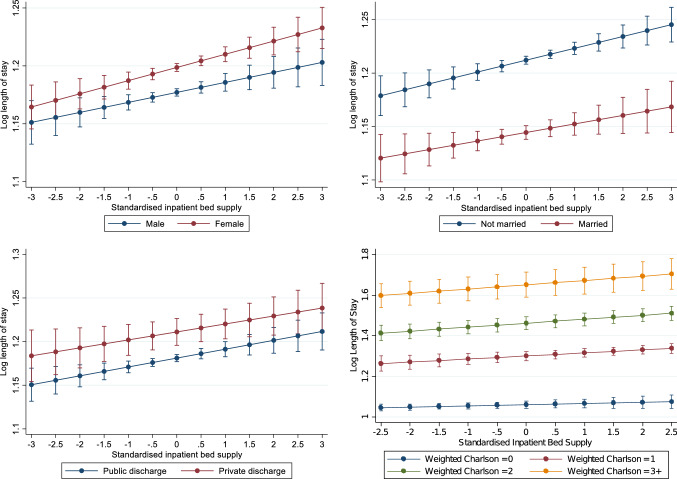
Determinants of length of stay for emergency inpatient discharges (hospital-level model): 2010–2015

Figure 4 also shows that hospitals with a greater number of medical card admissions and sicker patients (as measured by the average number of diagnoses per admission) have longer length of stay. Larger hospitals tend to have shorter length of stay once more.

Inpatient bed supply may have differing effects on length of stay across patients with different characteristics. In Fig. 4, we interact inpatient bed supply with sex, marital status, private discharge status and weighted Charlson comorbidity score to test whether reductions in inpatient supply may reduce length of stay at a greater rate for females (versus males), married patients, private patients or sicker patients with more comorbidities. Results are based upon the linear regression model from column III in Table 3. Overall, we find that there is little heterogeneity in the effect of inpatient bed supply on inpatient length of stay in the groups examined.

## Discussion

The changes in inpatient length of stay in Ireland between 2010 and 2015 were closely related to changes in bed supply that occurred during those years. Descriptively, there was a U-shaped pattern in average length of stay during our sample period that corresponds with a similar pattern in inpatient bed supply. In each of the regression analyses, controlling for a range of pertinent discharge-level characteristics and discharge case-mix, we find a positive, statistically significant relationship between length of stay and bed supply; a higher level of bed supply is associated with longer length of stay. The results are consistent across model specifications and when length of stay is measured at the individual discharge or hospital–month level. We standardise the measure of bed supply so that the models focus on the relative availability of beds across hospitals rather than the absolute levels. This is necessary because hospitals vary considerably in size within our sample. To the extent that length of stay is affected by the interaction of demand and supply, and the demand facing each hospital is likely also affected by its scale, it seems appropriate to estimate these models using relative metrics.

To illustrate the magnitude of the effects, we use the changes in average length of stay between 2010 and 2012 as points of reference, which equates to the period where the most severe public expenditure cuts were experienced. The findings predict that approximately 40–60% of the reduction in emergency inpatients’ length of stay observed during this period may have been a result of bed supply reductions experienced in those years. Examining heterogeneous effects, the positive relationship between length of stay and inpatient bed supply is observed across sexes, marital status, public/private discharge status, and for patients with differing levels of comorbidities.

These results show that the link between length of stay and bed supply can flow in both directions. This is an important finding given that length of stay is used as a key measure of efficiency within the hospital sector. However, interpreting changes in length of stay as indicating efficiency effects without understanding the context could be counterproductive. As we show, length of stay reductions can be driven by rationing or restraining supply, and that in periods where expenditure cuts or systems changes occur, lower length of stay may be (in part) indicative of the lack of resources or bed supply available as opposed to reduced demand for care or shifting of some care to other settings. Other countries have seen reductions in bed supply and increases in occupancy rates in recent years (Appendix Table A1), some of which are due to healthcare expenditure cuts. In the English NHS, for instance, in recent year acute bed numbers have fallen. This has coincided with increases in occupancy rates; 87% in 2010/11 to almost 92% at the end of 2018 [38, 39]. Therefore, where bed occupancy is over the 85% recommend maximum level, and in many cases approaching 100%, investing in bed capacity is a necessity regardless of average length of stay reductions that may be observed.

There is evidence that at the onset of economic crises, many governments, including in Ireland, initially enact a counter-cyclical policy by expanding healthcare expenditure in response to recession [25]. But in Ireland, any initial counter-cyclical measures were subsequently replaced by severe cuts to expenditure. Previous analyses of the Irish healthcare system during the economic crisis have argued that efficiencies were seen in the public hospital system at the beginning of the recessionary period (2008–2012), with hospitals “doing more” (inpatient and day-case activity) “with less” (reduced budgets) [23]. The authors acknowledge that the continued lack of staffing resources and capacity did result in lower activity and increased waiting lists post-2012 [23]. Results in this study show that while hospitals reduced length of stay as bed supply contracted, the increase observed in inpatient activity may be at least in part a result of insufficient  bed supply in the first place. Subsequent reductions in elective admissions and increases in waiting numbers for elective treatment (Appendix Figures A3 and A4) also point to this.

The implications of low bed supply are likely to go beyond lower length of stay and affect patient outcomes more generally. This has been most acutely seen in the recent COVID-19 pandemic. Evidence showed Ireland had amongst the lowest hospital bed supply, and intensive care bed supply, compared to European peers, at the beginning of the crisis [40]. This in part resulted in private hospitals being nationalised, reductions in elective surgeries (and increases in waiting numbers), and a much greater emphasis to increase hospital capacity [40]. The low levels of bed supply are also one of the main reasons for Ireland having some of the most restrictive COVID-19 lockdowns in Europe [41].

The lack of a unique patient identifier in Ireland makes it difficult to examine outcomes such as mortality and readmissions. However, previous studies internationally have found shorter inpatient length of stay often results in greater readmission rates [42, 43]. Recent evidence from Denmark and the UK shows that higher bed occupancy rates, closely related to bed availability, were associated with higher mortality rates [44, 45]. Given that Ireland has the highest inpatient bed occupancy rates in the EU, these latter findings are worrying, and worthy of further investigation.

Lower bed supply is also likely to impact elective care and contribute to waiting lists. Private hospitals provide approximately 15% of all inpatient care in Ireland [33], with the majority of this care being elective or less complex emergency care. The lack of information on private hospital care means we could not explicitly examine elective care in this work. However, Figure A3 in the Appendix shows that over the time period examined in this study, the number of elective discharges has actually fallen, despite the increasing and ageing population during this period. In 2010, 24% of inpatient discharges were elective, while only 19% were in 2015. There is little doubt this has contributed to increases in waiting lists for elective care, and waiting times, as shown in Appendix Figure A4. Waiting lists in Ireland are much higher than comparative countries [46].

Across all regression analyses, we clustered standard errors at the level of the hospital. However, because standard errors are estimated at the group rather than at the individual level, the asymptotic assumptions may not hold when the number of clusters is small i.e. *n* = 26 for Tier 1 ED hospitals. There is evidence that a small number of clusters (less than 50) may be insufficient to estimate accurate standard errors [47], and in this instance the standard errors may be quite large. In the findings, the relationship between length of stay and bed supply was statistically significant in general. However, as only a small number of public hospitals exist in Ireland, and clustering is necessary due to differences in characteristics across hospitals, the problem of clustering is likely to be a continued feature of analysis of hospital care in Ireland.

## Supplementary Information

Below is the link to the electronic supplementary material.

Supplementary file1 (DOCX 37 KB)

## Data Availability

This study uses hospital discharge data provided by the Healthcare Pricing Office (HPO) that are subject to data protection rules and the data cannot be made publicly accessible. Please contact the HPO for data access.

## Footnotes

1 There is evidence that initially many EU countries enacted a counter-cyclical policies by expanding healthcare expenditure in response to recession [25].

2 Changes to staffing levels and skill-mix, greater staff flexibility and increases in hours worked also occurred [24].

3 This information is available from the Health Service Executive Performance Reports 2008–2012.

4 Diagnosis Related Groups are a statistical system of classifying inpatient stays into homogenous groups for the purposes of payment. Similar to HRGs in HES.

5 Month of discharge, or season of discharge are not included as bed availability is a linear combination of the month effects, and therefore it is inappropriate to include both measures simultaneously [35].

6 The negative binomial model outperforms Poisson when examining these data as Poisson performs poorly in modelling length of stay overdispersion, especially where data is clustered for example at the hospital level.

7 Overnight emergency inpatient length of stay, which encompasses more patients than in this study’s analysis, was 7.7 days in 2016, 7.8 days in 2017 and 7.9 days in 2018.
