# Supplementary material for: The impact of inpatient bed capacity on length of stay
Source: Eur J Health Econ. 2021 Sep 4;23(3):499–510. doi: 10.1007/s10198-021-01373-2 (PMC8417615; doi:10.1007/s10198-021-01373-2)
Supplement: Supplementary file 1 — Supplementary file1 (DOCX 37 KB) [file 10198_2021_1373_MOESM1_ESM.docx]

# Appendix

Figure A1: Acute Inpatient Bed Numbers per 1000 people across OECD: 2005 - 2014

|  |
| --- |

**Source**: OECD. Health at a Glance 2016

Table A1: Inpatient Bed Occupancy Rates in Europe: 2010-2015

|  | **2010** | **2011** | **2012** | **2013** | **2014** | **2015** |
| --- | --- | --- | --- | --- | --- | --- |
| Ireland | 91.4 | 91.9 | 92.6 | 93.8 | 93.3 | 94.7 |
| UK (England)**^†^** | 86.6 | 86.9 | 87.6 | 87.5 | 88.5 | 89.0 |
| Switzerland | 87.5 | 88.8 | 82.9 | 83.6 | 82.6 | 83.3 |
| Malta | 81.5 | 83.2 | 83.2 | 80.7 | 81.8 | 81.7 |
| Norway | 85.6 | 83.7 | 84.8 | 83.8 | 82.8 | 80.4 |
| Germany | 79.0 | 79.0 | 79.2 | 79.3 | 79.7 | 79.8 |
| Italy | 78.7 | 78.5 | 77.5 | 77.3 | 77.6 | 78.9 |
| Croatia | 75.2 | 76.7 | 77.3 | 73.7 | 71.7 | 76.3 |
| Spain | 76.4 | 75.4 | 75.8 | 75.8 | 75.7 | 75.8 |
| Austria | 77.0 | 76.3 | 75.2 | 75.4 | 75.0 | 74.3 |
| Czech Republic | 73.8 | 72.8 | 73.1 | 73.9 | 74.9 | 74.3 |
| Lithuania | 72.0 | 73.1 | 72.5 | 71.6 | 72.8 | 72.2 |
| Cyprus | 84.2 | 90.9 | 75.8 | 74.4 | 74.7 | 72.2 |
| Luxembourg | 72.5 | 72.4 | 73.2 | 71.6 | 71.1 | 71.6 |
| Latvia | 71.1 | 70.4 | 68.1 | 68.0 | 69.7 | 70.7 |
| Hungary | 71.6 | 71.1 | 69.2 | 70.4 | 70.8 | 69.3 |
| Slovenia | 69.7 | 68.9 | 68.8 | 67.9 | 68.4 | 68.8 |
| Slovakia | 66.5 | 65.5 | 67.3 | 67.4 | 68.9 | 68.7 |
| Serbia | 70.9 | 69.7 | 68.5 | 70.2 | 68.3 | 67.6 |
| Estonia | 70.8 | 71.0 | 69.1 | 69.4 | 69.1 | 67.0 |
| Liechtenstein | 74.7 | 68.8 | 69.6 | 77.5 | 65.5 | 65.4 |
| Portugal | 64.3 | 63.6 | 66.2 | 64.2 | 64.8 | 64.0 |
| Belgium | 78.2 | 78.0 | 78.4 | 79.6 | 78.4 |  |
| Greece | 70.6 | 72.3 | 73.6 |  |  |  |
| France | 75.0 | 75.0 | 75.0 | 75.8 | 75.1 |  |
| Netherlands | 52.8 | 47.5 | 45.6 |  |  |  |

Source: Eurostat, **^†^** NHS England (2017) <https://www.england.nhs.uk/statistics/statistical-work-areas/bed-availability-and-occupancy/bed-data-overnight/>

Figure A2: Emergency Admission Length of Stay Distribution: 2010-2015

|  |
| --- |

Figure A3: Percentage of Inpatient Discharge by Emergency or Elective Admission: 2010-2015

|  |
| --- |

Source: HPO HIPE Reports 2010-2015

Figure A4: Number of Cases on Elective Inpatient Treatment Waiting List: June 2010-2015

|  |
| --- |

Source: NTPF
